# Programmes for pathology: improving health outcomes for low- and middle-income countries in the artificial intelligence paradigm

**DOI:** 10.1093/inthealth/ihaf036

**Published:** 2025-04-15

**Authors:** William Matupi

**Affiliations:** University of Nottingham Medical School, Queens Medical Center, Lenton, Nottingham NG7 2UG, UK

Artificial intelligence (AI) is on the precipice of revolutionising our world. Our societal, economic and political problems are becoming increasingly technological.^1^ Within healthcare, AI can digest vast amounts of data, using sophisticated algorithms to find statistical patterns beyond that of even the most experienced clinician.^2^ This emerging paradigm offers a unique opportunity for healthcare systems in low- and middle-income countries (LMICs) and has the potential to save lives. However, while AI will no doubt improve health outcomes in LMICs, this commentary argues it cannot be viewed as a panacea. In the *Genealogy of Morals*, Nietzsche proposes that scientific advancements cannot proceed philosophical, and therefore political, solutions. As a corollary, AI should not be viewed as a solution in isolation but work in tandem with a global political system that is designed to generate better outcomes for all.

Health outcomes in LMICs have improved, with drops in child mortality, infectious diseases like malaria and HIV, and vaccine-preventable illnesses such as measles, diphtheria and pertussis, alongside a reduction in maternal mortality. However, significant challenges remain.^3^ For example, 50% of the globe still does not have access to essential healthcare and 94% of maternal deaths still occur in LMICs.^4^ Furthermore, 70% of the world does not have access to safe surgery.^5^ Paul Farmer, co-founder of Partners in Health (PIH), proposes the strengthening of healthcare systems hinges on four pillars: staff, stuff, space and systems.^6^ Each of these elements is important in generating long-term and equitable outcomes. This commentary will first explore the many successes AI has had in mitigating challenges in each of these domains, and will then go on to discuss how to fully exploit the benefits of AI; the fields of technology, medicine and politics should not be viewed as disparate, but rather as interconnected disciplines, working together for meaningful change.

## Staff

To improve healthcare outcomes, sufficient appropriately trained staff must be distributed in locations that match a population's needs.^6^ The WHO estimates that by 2030 there will be a global shortfall of 10 million healthcare staff, with most of this burden being carried by LMICs.^7^ Underfunding for the training of healthcare professionals, difficulties in deploying workers to rural settings and the migration of skilled workers to high-income countries (HICs) are some of the reasons why this supply side scarcity has occurred.^8^

For example, one area facing significant scarcity is diagnostic imaging.^8^ In Kenya, 74% of the population live in low density rural areas; conversely, 76% of radiologists work in urban areas, with 90% working in major cities.^9^ This picture reflects much of east Africa. For instance, in Uganda, 56% of urban inhabitants receive necessary scans; however, in rural areas this is as low as 10%.^10^ This shortfall of trained staff and infrastructure becomes even more critical when one considers the importance of chest radiology in identifying pathology such as TB, chronic obstructive pulmonary disease and malignancy.^11^^-^^13^

AI has been used to decentralise and automate imaging results, reducing the reliance on scarce expertise.^8^ In 2017, Rology, set up by a team of radiologists in Egypt and Kenya, created a platform to bridge healthcare facilities to a cloud where images can be uploaded and interpreted by AI to generate information to aid diagnosis.^14^ Moreover, images can be reviewed by radiologists worldwide to offer their interpretation. Rology has already shown promising results, partnering with 150 hospitals across nine countries in Africa and the Middle East, already claiming to have saved 700 000 lives.^15^ These promising results show that with the use of AI technology, labour scarcity can be challenged in LMICs by automating the critical tasks required to obtain an accurate and timely diagnosis for patients.

## Stuff

Although the notion appears rudimentary, healthcare systems need the right ‘stuff’ to function.^6^ In LMICs, public sector accessibility to essential medicines is poor, with provision reaching 38–40% of the population's needs.^16^ Furthermore, within the surgical specialty, 70% of operating theatres globally do not have access to a pulse oximeter, a basic requirement for monitoring patients under anesthesia.^5^ While some researchers acknowledge the benefits of AI in finding new treatments for disease,^17^ the aforementioned evidence suggests significant marginal gains can be achieved by using AI to better utilise existing resources.

Due to increasing urbanisation, ageing populations and lifestyle changes, one of the growing challenges for LMICs is dealing with malignancy.^18^ According to the International Agency for Research on Cancer, in 2022, of the recorded 20 million new incidences of cancer that emerged globally, lung cancer was the most prevalent, totalling 12.4%.^17^ Furthermore, 85% were attributed to non-small cell lung cancers (NSCLC).^19–21^ LMICs bear a high burden of these cases, with 57.3% presiding in Asia.^22,23^ Moreover, due to the specialist and scarce nature of cancer medication, the cost of managing patients is high. Research by China's National Cancer Center suggests that in 2019 national cancer expenditure exceeded 220 billion yuan (US$30 billion).^24^

AI has been shown to help effectively allocate scarce medications. Chang et al. developed an AI model to determine the effectiveness of NSCLC medications based on malignancy size and the presence of tumour markers.^25^ Using this information along with the estimated cost of each treatment, they have created a treatment–cost ratio for each drug, allowing the management of NSCLC to be allocated more efficiently across China. A model, they propose, that can be expanded across a variety of developing countries.

Another area in which AI has shown promise is within the field of immunisations, the most cost-effective public health intervention.^26–28^ One of the global challenges of intervention is stagnating coverage rates in sub-Saharan Africa. Rates of coverage for third doses of pertussis, diphtheria and tetanus have remained at around 72% since 2010.^29,30^ One of the reasons for this plateau is the lack of data usage to inform stock management of vaccines.^31,32^ Macro-Eyes uses machine learning (ML) to predict future vaccine uptake across two regions in Tanzania.^33^ Combining data from 710 health care facilities across the provinces of Arusha, Tanga and Kilimanjaro, along with additional data from the Tanzania Health Facility Registry, the Tanzania National Bureau of Statistics and an online GPS visualiser, the ML tool has been able to provide the first forecasting model to predict vaccine utilisation that can be applied to many countries. This tool provides programme managers with access to quality information on stock demand in a specific location, improving supply side limitations of vaccine uptake.^34^ Both examples are key in highlighting the potential for AI in LMICs. If stocks can be better managed, more patients can be treated through accurately equating supply with demand.

## Space

Safe well-equipped spaces are vital for patients to be treated effectively.^6^ This is a particularly significant challenge in relation to global migration, which remains a major challenge for LMIC health systems.^35^ The latest available data estimate that global migration totalled 3.6% of the global population in 2020, with nearly one-half of migration flows being inter-regional among LMICs. Many healthcare systems have lacked resilience in dealing with such demand, finding it difficult to predict where best to prioritise building healthcare facilities. While most migration is related to work, family and study, a proportion arises from conflict, natural disasters and persecution.^36^ For example, in 2012, 450 Syrian refugees found themselves in the Jordanian desert fleeing the civil war in their home country. Within 24 h the Za'atari refugee was formed.^37^ Currently, the camp has 80 000 experiencing chronic and acute diseases, including hypertension, diabetes, upper respiratory tract infections and fractures.^38^

One of the challenges in the camp is finding safe accessible spaces to build healthcare facilities.^39^ The Environmental Systems Research Unit, in combination with the United Nations High Commissioner for Refugees, has sought to solve this dilemma by pioneering the implementation of AI in combination with bottom-up community activism.^40^ The RefuGIS programme educates community members on the concepts of using computer programs to map the camp, focusing on skills such as using coordinate systems, thematic mapping and graphic design.^41^ Esri then took this geospatial information and used AI to generate data to inform community decision-making on the optimum location for healthcare centres and offices.^42,43^

What is perhaps most remarkable about this example is the dual effect of informing healthcare decision-making and empowering a community. In the book *While The Earth Sleeps We Travel*, Ahmed Badr states the word ‘refugee’ is associated with both an ending and a beginning, but the middle is often ignored.^44^ The use of AI not only to inform decision-making, but also to give dignity and autonomy to potentially vulnerable patients, demonstrates the powerful scope of the prospects of AI.

## Systems

Healthcare systems do not operate in a vacuum. One of the most critical areas in improving health outcomes for LMICs is ensuring health infrastructure works effectively within wider systems. This includes good leadership and robust financial systems, along with strong social support.^6^

COVID-19 had a devastating effect on many people living in Togo. Prior to the pandemic poverty had been declining. However, due to the impact the disease had on employment, particularly the informal sector (which employs 78.3% of the total workforce), the poverty rate increased to 46.2% in 2020. Moreover, by this year, 22% of the population required humanitarian assistance and the number of chronically malnourished children aged <5 y was 23.8%.^45^

One of the challenges the Togolese government faced was accurately targeting the individuals with the lowest income to optimise the distribution of emergency financial aid. The last census was conducted in 2011. In addition, the data collected were insufficient to identify those in most need of emergency cash. In response to this the government, in collaboration with the World Bank, used deep-learning algorithms to identify the 100 poorest Cantons in the country. Large-scale phone surveys of 10 000 individuals were conducted to identify the truth of what people were facing on the ground. ML algorithms were then used to predict the consumption of 70% of the population.^17,46^ Using these algorithms the government could provide direct cash transfers to the poorest. As a result, evaluation of the Novissi programme was found to have a statistically significant impact on food security and mental health.

In 2020, global figures suggested 9 million people died of food insecurity, generating a high burden on healthcare systems.^47^ The use of AI to manage food security is one important way in which the technology can be used to indirectly improve healthcare systems.

## Looking forward

At the beginning of this commentary, I proposed that many of our human problems are becoming technocratic ones. This is the antithesis to Nietzsche's work in the *Genealogy of Morals*, where he states collective values for approaching human problems are first needed in which science can then serve. Moreover, Nietzsche states that science can never autonomously create its own values.^48^ In line with this, AI can be considered the guiding hand; however, economic and political incentives will ultimately be the rate determining step as to the effectiveness of AI in such settings. Barriers to the implementation of AI include access to digital infrastructure, education of staff and regulation of data.^49^ For sovereign governments to have effective autonomy over the use of AI in their healthcare systems, both neoclassical and Keynesian economics assume a prerequisite of robust political institutions and well-functioning markets.^50,51^ Therefore, countries lacking these criteria are likely to see insufficient domestic and international investment in the components necessary to maximise the benefits of AI, potentially broadening inequalities between HICs and LMICs.

In conclusion, it is evident that AI has the potential to drastically improve health outcomes across LMICs in all four categories discussed; however, ultimately such a dilemma is not just a technocratic issue but a human one. For this remarkable technology to justly serve those across all areas of society it is important it is not viewed as a panacea. AI should work in tandem with an understanding of the human responsibility required to create a global system that facilitates better outcomes for all. In the absence of such political change, with or without AI, global development remains as complex as ever.

## Data Availability

No new data were generated or analysed in support of this research.
